# 3D metal lattice structure manufacturing with continuous rods

**DOI:** 10.1038/s41598-020-79826-6

**Published:** 2021-01-11

**Authors:** Bashir Khoda, A. M. M. Nazmul Ahsan, Abu N. Shovon, Adeeb I. Alam

**Affiliations:** 1 Department of Mechanical Engineering, The University of Maine, 5711 Boardman Hall, Orono, ME 04469-5711 USA; 2 School of Engineering and Technology, Western Carolina University, 389 Centennial Dr., Belk 221, Cullowhee, NC 28723 USA

**Keywords:** Mechanical engineering, Mechanical properties

## Abstract

In this paper, a new possibility of fabricating a metal lattice structure with a continuous rod is demonstrated. A multi-layer, periodic, and aperiodic lattice structure can be manufactured with a continuous thin rod by bending it with a repetitive pattern. However, joining their nodes are challenging and an important problem to solve. This paper is investigating the joining of nodes in a loose lattice structure by delivering materials through the dipping process. Both liquid state (epoxy) and solid-state (inorganic particles) joining agents are considered for polymer–metal and metal–metal bonding, respectively. Liquid Carrier Systems (LCS) are designed considering their rheological behavior. We found 40% solid loading with the liquid carrier system provides sufficient solid particles transfer at dipping and join the lattice node using transient liquid phase bonding (TLP). 3D metal lattice structures are constructed, and their mechanical properties are investigated. The lattice structure shows comparable strength even with smaller relative density (< 10%). The strength and elastic modulus of all the fabricated samples decreases with the increase in cell size, which is consistent with the traditional wisdom.

## Introduction

Lattice structures are compelling candidates for the development of a wide range of engineering structures, i.e., boat hull, propellers, airplane wings, fuselage, biomedical implants and prosthetics, automobile chassis, heat exchangers, shelters, and bridge structures, etc. The open cell structures consist of a number of repetitive connected members or tessellated unit cells which are constructed with elements/struts^1^ and connected through point contact, often defined as a node. Assembling these unit cells forms a complex structural network^1^ and follows Maxwell’s criterion^2^ which helps to determine the strut-based topologies to equilibrate external force and moment. Higher nodal connectivity (> 6) in 3D lattice is needed for structural rigidity^3^ (i.e., stiff and strong structures), while compliant and consistent deformation may result from bending-dominated structures^4^. Superior and predictable performance in lattice structure requires design and manufacturing perfections (i.e., unit cell parameters, connected nodes, porosity, etc.). Fabrication imperfections, namely topological (variations in nodal connectivity and missing strut)^5,6^, and dimensional (variations in cellular dimensions)^7–9^, are widespread challenges even in mesoscale (couple mm) unit cell size lattice, which makes the lattice structures often unattainable. Additional to the manufacturing challenges of the 3D lattice structure includes their fabrication speed, topology design, large data feed, and shape conformity, which are some non-trivial issues.

**Figure 1:**
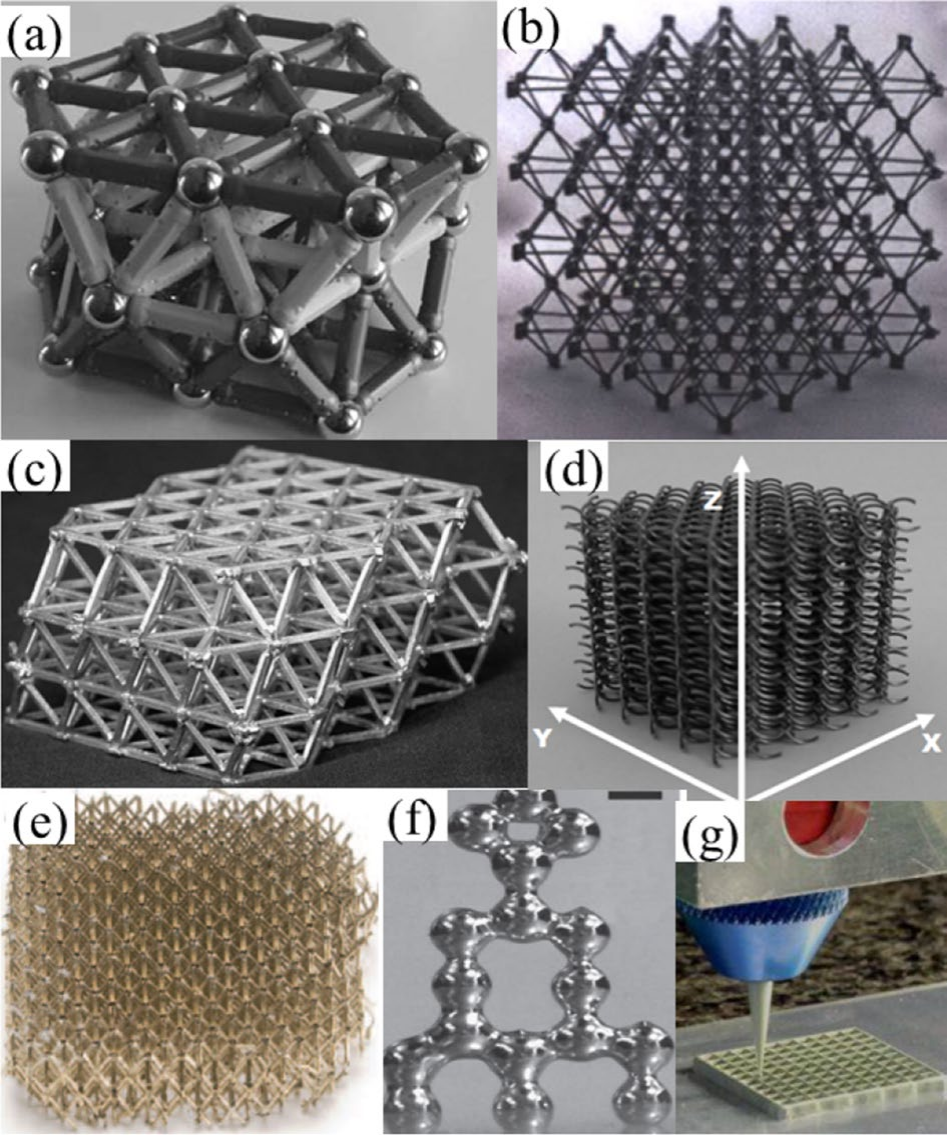
Multilayer lattice structure manufacturing technique (**a**–**b**) lattice (macro scale) building block assembly^10^; (**c**) multi-stage manufacturing of pyramidal lattice sandwich^11^; (**d**) Strucwire/Wire-woven with 0.63 mm steel wire^12^; (**e**) Ultralight micro-lattice (5 cm length) with electroless nickel plating^13^ (**f**) liquid metal drop 0.5 mm scale^14^ (**g**) direct write 200 µm nozzle^15^.

Traditional lattice manufacturing processes (some are shown in Fig. 1) are complex and costly due to their intricate architecture and nodal connections. Commonly used open-cell lattice structure manufacturing techniques are: investment casting^16^; robocasting^17^; wire-woven^18^; high-temperature forming and diffusion bonding^19^; electroless nickel plating^13^; sacrificial mold coating^20^; interlocking grid assembly^21,22^; lattice block by stacking^23^. Disconnected or unbounded nodes are common in multi-stage lattice manufacturing processes^24^. For example, due to the rheological constraint of the molten metal, early solidification, underfill of the thin and twisted sections of complex lattice structure is reported in literature^25^. Aqueous or polymer-based metallic inks are designed with high solid loading for robocasting. The subsequent sintering (consolidation) process resulted in pores, cracks, and shrinkage, which can be prevalent in thin section^26^. A contemporary shape-changing 4D printing technique has demonstrated the potential to manufacture 3D objects from 2D or planner patterns^27^. Predetermined external stimuli such as heat, water, light, pH are used on the active composite material, which then evolves by following the shape memory effect^28^. The concept has been demonstrated for flexible electronics circuit^29^, actuator, elastic hinge, end-effector gripper for soft robotics^30^.

Recently, metallic lattice fabrication processes based on wires have been reported, called ‘wire-woven metals’^18,31^. In the wire-woven technique, a textile-based weaving process is used to manufacture lattice structures with repetitive symmetric geometries from metal wires. Typically wire segments are straightened and then preformed and/or crimped into a specific pattern creating a ‘textilecore’^32^. One of the successful applications for ‘textilecore’ is the industrial conveyor belt for a harsh manufacturing environment. 3D structures are also reported with preformed triangular wavy, trapezoidal, or helical patterns, following their spin insertion in different groups from multiple directions creating octet and kagome-2, and bulk diamond-like lattices structure. Individual wires are then joined using brazing, soldering, sintering, or adhesive bonding. While large size periodic topologies can be achieved with the wire-woven technique, multi-scale controlled heterogeneity and/or aperiodic cell type is not possible with this process. Also, multi-layer 3D shapes can be morphed by stacking, and subtracting woven pieces together, which will cause fractional unit cell at the boundary^33^. This may result in a lack of continuity along with 3D and shape conformity, which will cause degradation in their intended performance. Indirect application of wire-woven technique has also been reported for constructing hollow truss metallic porous microstructure^34^. Space holding template is first been built with the wire-woven technique using polylactic acid (PLA) wires, which is then infiltrated with UV-curable resin. Finally, nickel–phosphorus (Ni–P) thin-film through electroless plating is performed to construct the thin wall lattice structure.

In Contrast, direct-write or 3D printing^15^, often considered as a single stage digital manufacturing process with incremental consolidation of low dimensional forms of feed-stock materials, i.e., powder, liquid vet, or semi-molten filament to construct lattice structure following their virtual design^35^. ASTM classifies the available AM processes into seven categories and among them, extrusion process^36^, powder bed fusion^6,37,38^, and direct energy deposition are often used to fabricate complex structures, i.e., metal lattice. The extrusion-based process often called the direct ink writing technique, utilized robotic-assisted deposition to print lattice structures with aqueous or polymer-based metallic ink. Nozzle agglomeration, pores or microvoids, uneven surfaces, cracks, and shrinkage can be a challenge for lattice strut or thin section^39^. In powder bed fusion processes, the quality and performance of the structures often depend upon the use of heat sources, control in process parameters, and post-processing techniques. For example, a laser beam is used to melt the metal powder in the selective laser melting (SLM) technique. However, uncontrollable thermo-mechanical behavior^40^ in the tool-less 3DP process brings (a) diverse and amorphous (metastable) microstructure^5,37^ (b) higher contamination^41^; and (c) shrinkage and disconnected nodes^17^.

Alternatively, the electron beam melting (EBM) technique uses an electron beam in a highly controlled vacuum environment for melting and joining the metal powder. The process uses preheating of the powder to around 80% of the melting point using the same electron beam before it scanned at high power to melt the powder^42^. Due to the use of preheating, the overall heat transfer process is significantly more controllable, and for lattice structures, the thermal residual stress issue is also often considered negligible due to the high substrate temperature and the “stress-relieving effect” in-process. Thus, the EBM powder bed fusion technique could be advantageous in fabricating lattice structures. For instance, heat treatments to relieve residual stresses are not necessary in EBM as the powder bed is preheated in this process^43^. Additionally, internal porosity in EBM made parts can be reduced or eliminated with special heat treatments such as hot isostatic pressing (HIP)^44^. The fabrication of lattice structures using metal AM processes is also significantly different from the fabrication of bulk objects. Overall, the AM approach poses some substantial limitations to lattice structures such as surface quality^38,45^, resolution, low powder utilization and build defects, and geometric imperfections^9^. Many of these reported issues may often be resulted from the knowledge gap rather than intrinsic process limitations and can be eliminated by applying post-processing techniques^46,47^.

**Figure 2:**
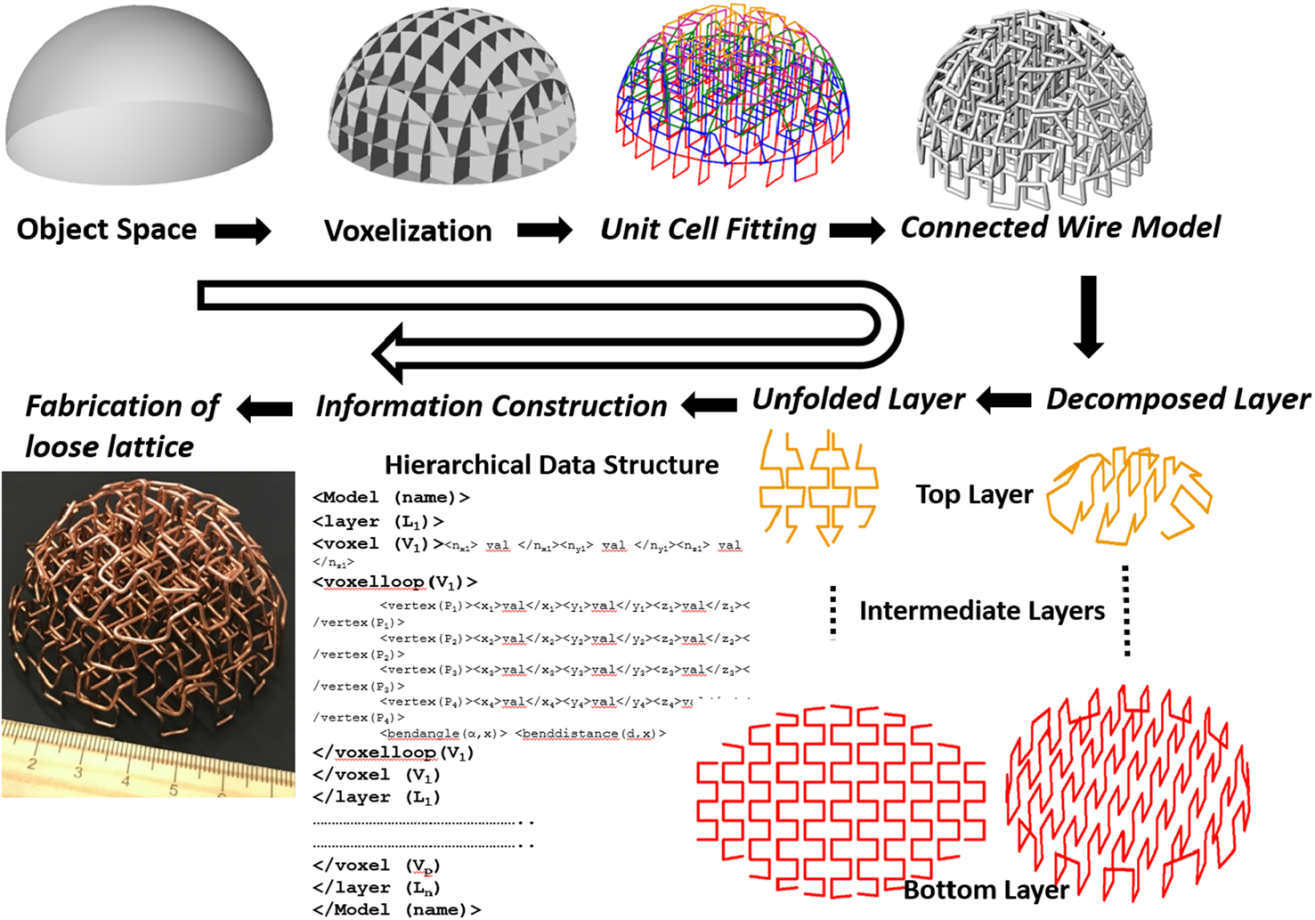
Construction of loose lattice structure with continuous wire bending technique.

This paper is investigating a new manufacturing process of a lattice structure with continuous rods. Fabricating lattice structures with 1D metallic wires have several advantages compared to other forms of material, i.e., powder, 2D sheet, liquid metal. They are easy to handle, radially available, cheap compared to other forms of metals, minimal waste, and homogeneous. Almost all metals, including zirconium and tungsten, can be formed into a wire^48^. A multi-layer, periodic, and aperiodic lattice structure can be manufactured with continuous thin rods by bending it with a repetitive pattern in a casual way. A more systematic approach is shown in Fig. 2, where the topology optimization technique from our prior work (‘*pixelization to voxelization’*^49–51^ is employed. A CNC machine is retrofitted to construct the designed 3D lattice structure with disjoined nodes, which is defined as a loose lattice structure in this paper. It should be mentioned that the sophisticated topology optimization technique is not mandatory for making the structure with wire material. However, the generated nodes are required to be welded to achieve mechanically robust functional structures. Due to the size of the unit cell and complex structure, the nodes are often inaccessible and cannot be welded with traditional processes i.e., spraying, arc, TIG/MIG, or gas welding. This paper is investigating the joining of nodes in loose lattice structure by delivering materials through dipping. The individual unit cells considered in this paper are meso- and macro-scale dimensions (from couple mm to several cms)^52,53^, whereas the dimension of the structure can be multiple order of magnitude larger. A polymer–metal adhesive bonding with an Epoxy solution and a metal–metal joining with transient liquid phase bonding (TLP) technique has been investigated here.

## Result

### Loose lattice joined with epoxy polymer (polymer–metal join)

**Figure 3:**
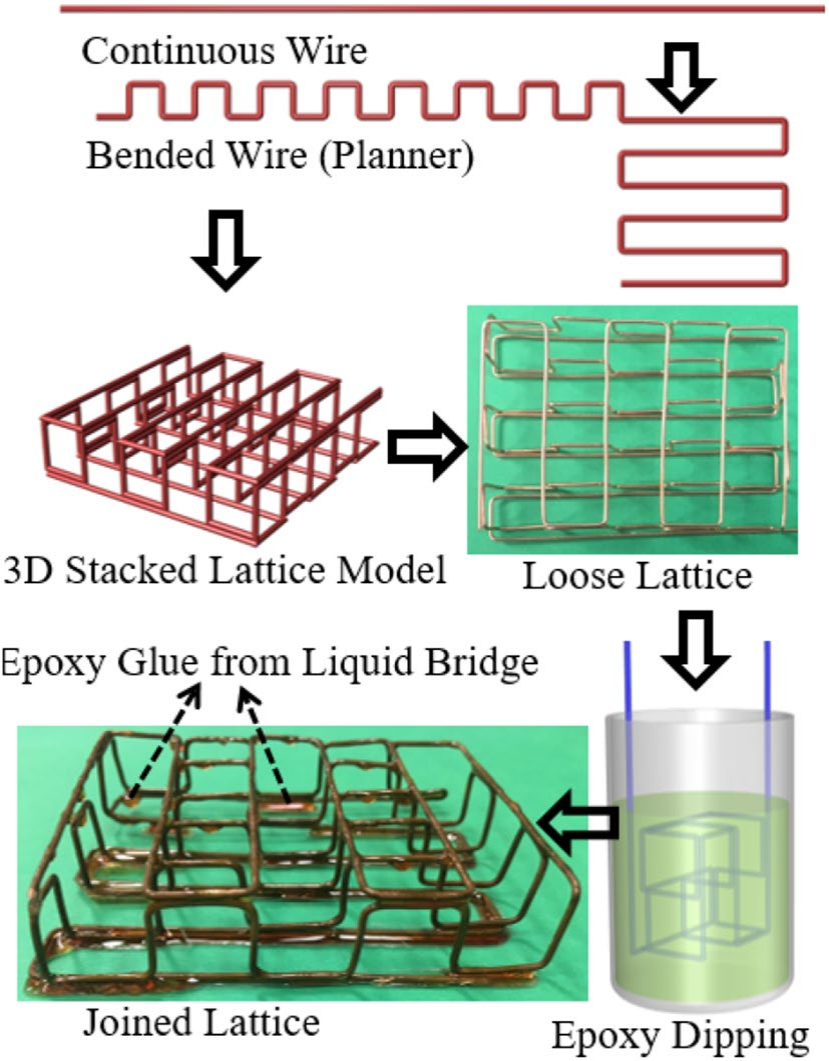
Sample lattice structure fabrication by joining the node with the epoxy polymer.

**Figure 4:**
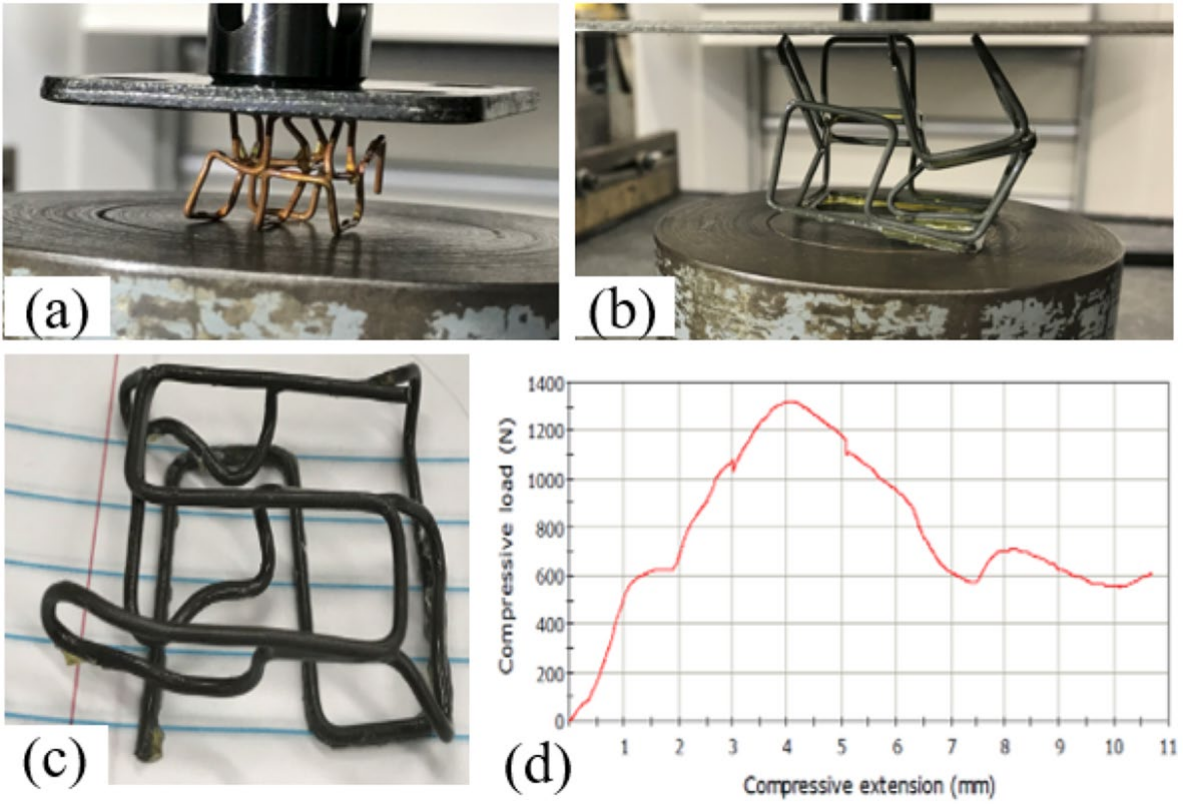
Compressive test of lattice structure with the continuous rod. The nodes are joined with 2000 Epoxy Resin and 2120 Epoxy Hardener (**a**) Copper wire Ф = 1.02 mm, cell size 5 mm; 2 × 2 × 2 structure (**b**) and (**c**) AISI1006 Carbon Steel wire Ф = 1.63 mm, cell size 15 mm with 2 × 2 × 2 and 2 × 2 × 1 structure respectively; (**d**) load profile for sample (**c**).

Commercially available 2000 Epoxy Resin and 2120 Hardener is used to connect the nodes of the loose lattice structure. The cylindrical metal substrate is immersed into the epoxy blend at a constant speed (0.1 mm/sec to 10 mm/s) and will remain there for a constant dwelling time of 10 s before retracting. No kinetic energy is applied during this dipping process. At the dwelling state, the polymeric chain will land on the metal surface, forming a polymeric layer due to the entropy advantage. The sticking energies per chain increase in proportion to the number of monomer units adsorbed on the substrate surface^54^. This formation is unavoidable when even weakly attractive surfaces come into contact with a polymer solution^55^. The wetting phenomenon will facilitate the adsorbed interface, and a liquid bridge will be formed between the adjacent surface, as shown in Fig. 3. The epoxy material is hardened at the curing stage and creating bonds at the node. Samples are fabricated and tested for mechanical properties, as shown in Fig. 4. As expected, the bonds can hold the node under a reasonable amount of load (Table 1). However, the failure (brittle fracture) occurs at the polymer–metal interface, which is the weakest point of the lattice structure. For better mechanical properties, metal–metal bonding is also designed and investigated in this paper.

**Table 1:** Properties of lattice structure nodes joined with the epoxy polymer.

Materials	Copper wire	AISI1006 carbon steel	AISI1006 carbon steel
Wire diameter (mm)	1.02	1.63	1.63
Cell size	2 × 2 × 2	2 × 2 × 2	2 × 2 × 1
Volume fraction	4.2%	6.2%	6.2%
Measured stress (MPa)	0.98	0.83	0.64

### Loose lattice joined with transient liquid phase (tlp) bonding (metal–metal join)

**Figure 5:**
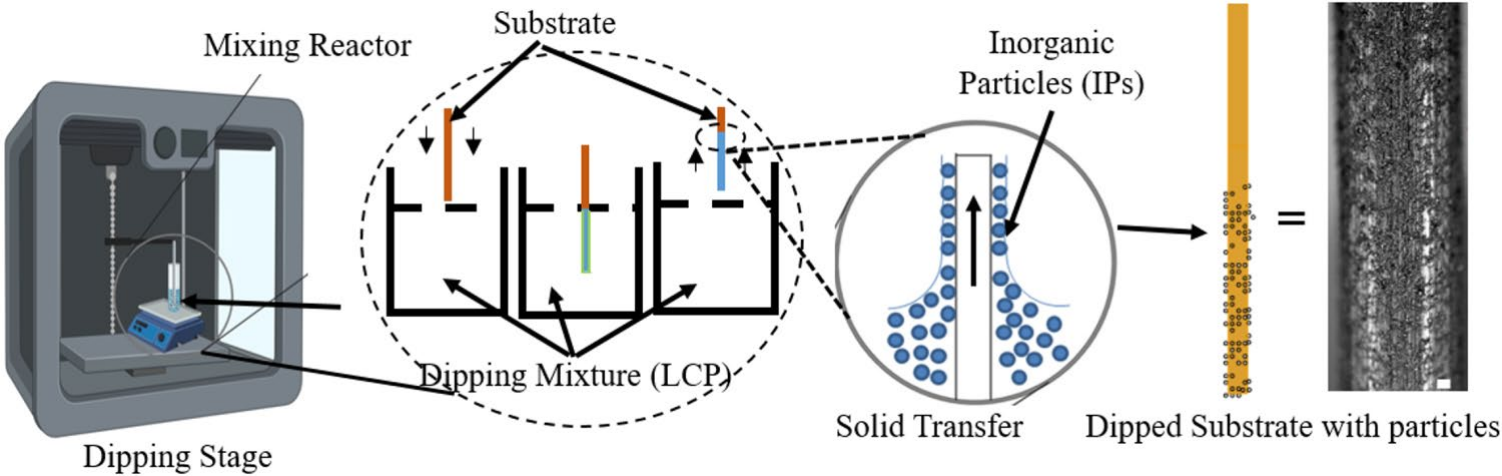
Metal substrate dipping and solid transfer process by entrapping IPs from LCP (micrograph) The scale bar in the rightmost figure is 100 µm.

**Figure 6:**
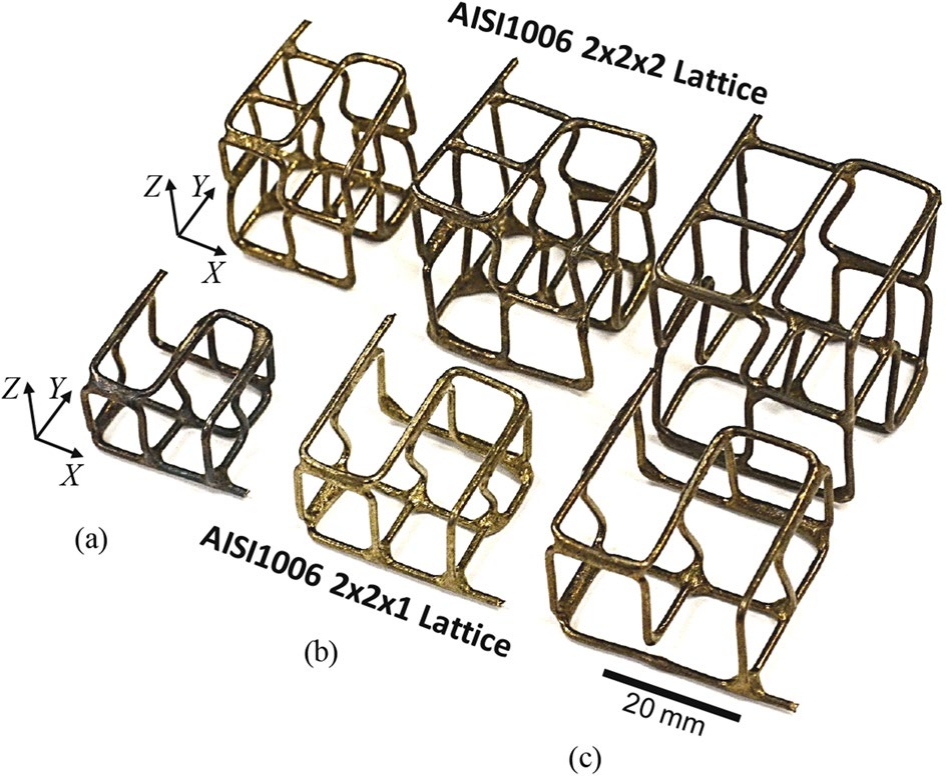
AISI1006 low carbon steel wire lattice structures fabricated and joined for compression testing. The measured relative density (**a**) 8.6% and 6.8% for 8 mm cell; (**b**) 6.4% and 5.3% for 10 mm cell; and (**c**) 5.0% and 3.8% for 12 mm cell with 2 × 2 × 1 and 2 × 2 × 2 structure respectively.

Transient Liquid Phase (TLP) bonding process is employed to join the struts at junctions by dipping the loose lattice into the interlayer alloy mixture, which is defined as a liquid carrier system (LCS). The interlayer alloy or inorganic particles (IP) are entrapped on the metal substrate due to the adsorption kinetics between the polymer glue, metal substrate, and inorganic particles (IP) as shown in Fig. 5. The interlayer alloy coated lattice structures are shown in Table 2, which are then transfer to a vacuum furnace at 10^–3^ torr. Sample lattice structures with AISI1006 material (diameter 1.02 mm) are designed and manufactured, as shown in Table 2 and Fig. 6. Both 2 × 2 × 1 and 2 × 2 × 2 cells with unit cell size 8 mm cell, 10 mm cell, and 12 mm can be seen in Fig. 6. To provide better nodal contact, some struts are extended by ± 1.5 mm, which caused some slanted vertical struts. The shiny color of the joined lattice can be attributed to the presence of Chromium in the interlayer alloy particles.

**Table 2:** Fabricated and joined cubic lattice structures.

	Loose lattice	Coated (green)	Joined with TLP
Cell size: 10 mm 2 × 2 × 1 cell lattice AISI1006			
Cell size: 10 mm 2 × 2 × 2 cell lattice AISI1006			
Cell size: 12 mm 2 × 2 × 1 cell lattice AISI1006			
Cell size: 12 mm 2 × 2 × 2 cell lattice AISI1006

Two groups of structures are fabricated and two samples are tested for mechanical properties: (i) single layer 2 × 2 × 1 cell lattice, which has two cells on both *X* and *Y* directions and one cell along *Z* direction; and (ii) double-layer 2 × 2 × 2 cell lattice, which has two cells on all directions. 8 mm, 10 mm, and 12 mm cell sizes as shown in Fig. 6. The nominal stresses ($\sigma_{lattice}$) of the lattice structures are measured by dividing the applied load by the initial cross-sectional area of the structures perpendicular to the load. The nominal lattice strains ($\varepsilon_{lattice}$) are measured from the deflection of the interface between the structures and the compression platens. The stress–strain curves obtained from the measured data are used to study the compression behavior of the sample structures.

**Figure 7:**
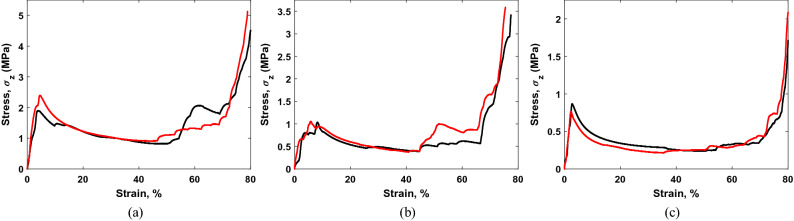
Stress vs lattice strain curves of 2 × 2 × 2 cell lattice structures for *Z*-axis loading: (**a**) 8 mm cell, relative density = 0.068; (**b**) 10 mm cell, relative density = 0.053; and (**c**) 12 mm cell, relative density = 0.038.

The stress $(\sigma_{lattice} )$–strain $(\varepsilon_{lattice} )$ behavior of the 2 × 2 × 2 cell lattice structures for the *Z-*axis loading can be observed in Fig. 7. The linear elasticity of all the samples spans over a very small strain range and the lattice elastic moduli $E_{lattice}$ are determined from this strain range. The linear elasticity ends through plastic failure at the plastic strain $\varepsilon_{lattice}^{pl}$ giving the lattice strength $\sigma_{lattice}^{{}}$. After plastic failure starts, the lattice structures show long plastic plateau regions until densification starts at the densification strain $\varepsilon_{lattice}^{D}$. Table 3 lists the values of strengths, plastic strains, and elastic moduli of the 2 × 2 × 2 cell structures for *Z-*axis loading. The lattice strength and elastic modulus decreased with the increase in cell size or reduced lattice relative density. The plastic strain also decreases with the increase in cell size except for the 10 mm cell, which can be attributed to the different behavior at the yielding region (Fig. 7b) compared to 8 mm and 12 mm cells. The stress–strain curves in Fig. 7 also indicate that the densification of all the 2 × 2 × 2 cell lattice structures starts at around 70% strain.

**Table 3:** Strength, plastic strain, and elastic modulus of 2 × 2 × 2 cell lattice structures for *Z-*axis loading.

	8 mm cell	10 mm cell	12 mm cell
$\sigma_{Z, \, lattice}^{{}}$ (MPa)	2.14 ± 0.25	0.93 ± 0.13	0.81 ± 0.05
$\varepsilon_{Z, \, lattice}^{pl}$ (%)	4.15 ± 0.46	4.62 ± 1.07	2.50 ± 0.21
$E_{Z, \, lattice}^{{}}$ (MPa)	1.48 ± 0.01	0.45 ± 0.13	0.36 ± 0.01

**Figure 8:**
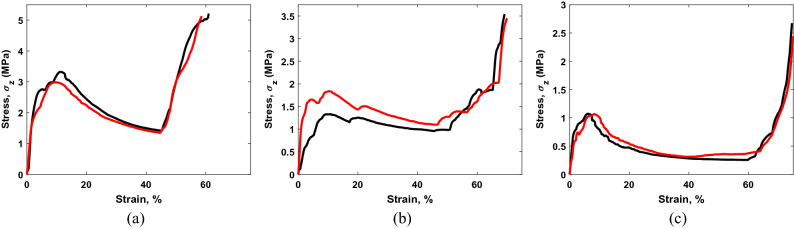
Stress vs lattice strain curves of 2 × 2 × 1 cell lattice structures for *Z*-axis loading: (**a**) 8 mm cell, relative density = 0.086; (**b**) 10 mm cell, relative density = 0.064; and (**c**) 12 mm cell, relative density = 0.05.

The stress $(\sigma_{lattice} )$–strain $(\varepsilon_{lattice} )$ curves for 2 × 2 × 1 cell lattice structures under *Z-*axis loading are given in Fig. 8. Table 4 lists the values of the strengths, plastic strains, and elastic moduli of the 2 × 2 × 1 cell structures for *Z-*axis loading. The lattice strength, plastic strain, and elastic modulus gradually decrease with cell size (reduction in lattice relative density). Additionally, it can be observed that the lattice strength, plastic strain, and elastic modulus for all cell sizes of 2 × 2 × 1 lattice are higher than that for all cell sizes of 2 × 2 × 2 lattice. This can be attributed to the increased relative density of the 2 × 2 × 1 lattices due to the additional top and bottom zigzag layers. The stress–strain curves in Fig. 8 also indicate that the densification of the 8 mm, 10 mm, and 12 mm cell 2 × 2 × 1 structures starts approximately at 45%, 55%, and 63% strain levels, respectively, which are lower than the densification strains of 2 × 2 × 2 structures. The increased relative density of the 2 × 2 × 1 structures again results in such early densification.

**Table 4:** Strength, plastic strain, and elastic modulus of 2 × 2 × 1 cell lattice structures for *Z-*axis loading.

	8 mm cell	10 mm cell	12 mm cell
$\sigma_{Z, \, lattice}^{{}}$ (MPa)	3.15 ± 0.17	1.58 ± 0.26	1.07 ± 0.00
$\varepsilon_{Z, \, lattice}^{pl}$ (%)	10.34 ± 1.07	9.46 ± 0.47	7.10 ± 1.05
$E_{Z, \, lattice}^{{}}$ (MPa)	1.72 ± 0.02	0.81 ± 0.47	0.51 ± 0.14

**Figure 9:**
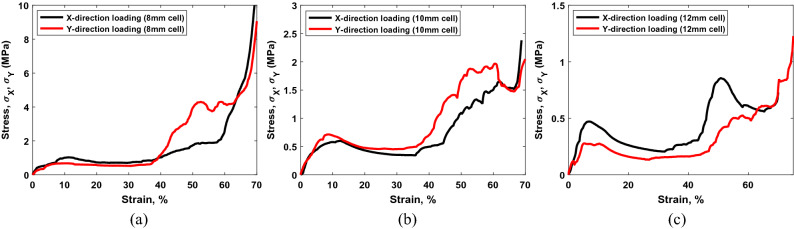
Stress vs lattice strain curves of 2 × 2 × 2 cell lattice structures for *X* and *Y*-axis loadings: (**a**) 8 mm cell, (**b**) 10 mm cell, and (**c**) 12 mm cell.

The 2 × 2 × 2 cell lattice structures are also tested under *X* and* Y* direction compression loadings to observe any mechanical anisotropy. The stress–strain curves demonstrate that the mechanical behavior of the structures along *X* and *Y* directions closely follows each other. However, the strengths ($\sigma_{X, \, lattice}^{{}}$, $\sigma_{Y, \, lattice}^{{}}$), plastic strains ($\varepsilon_{X, \, lattice}^{pl}$, $\varepsilon_{Y, \, lattice}^{pl}$), and elastic moduli ($E_{X, \, lattice}^{{}}$, $E_{Y, \, lattice}^{{}}$) along *X* and *Y* directions are lower than that along the *Z*-direction, as shown in Fig. 9. This indicates some mechanical anisotropy in the fabricated structures, which is also justified by the unit cell geometry. The unit cell designed for continuous bending path has more vertical struts than horizontal ones, making the structure stronger along vertical (*Z*) direction.

## Discussion

In this paper, we demonstrate a new possibility of fabricating metal lattice structures with continuous rods. The focus of this paper is to investigate the way to connect the nodes of the complex lattice structure that are hard to reach. The out of plane bent (3D) structure (loose lattice) will have nodes with a gap, which bring an additional level of complexity into their joining. Both liquid state (epoxy) and solid-state (inorganic particles) joining agents are considered and their delivery is ensured using the dipping process. Thus, the rheology controlling approach is used to design a liquid carrier system. In the case of epoxy, mixing the resin and hardener constructs a homogeneous solution and the rheology can be controlled with a mixing ration. However, in metal–metal TLP bonding, interlayer alloy need to be delivered, which are hard, spherical, large size, and high-density particles. Adding particles to any liquids modifies its physical properties, i.e., density, viscoelasticity. Liquid Carrier System (LCS) is the particle bearing mixture and display complex rheological behavior with the added fraction of IP. Added particles act as obstacles to the fluid flow, increasing the flow resistance or viscosity. A liquid carrier delivery system is designed with close to 40% solid loading. It is used to deliver the inorganic particles to join the nodes using the TLP process (details in the “Method” section).

For TLP bonding, the effective viscosity of our LCS is the pivotal parameter in determining the performance of solid particle transfer. The common dipping mixtures are often embedded with organic or inorganic fillers, nanoparticles, or clusters (*d* < 30 nm) that remain suspended by density-matching rheology^56^. The particle–particle and particle–surface adhesion force can be categorized as (i) electrostatic, (ii) van der Waals, and (iii) gravitational forces^57^. For submicron size particles, the electrostatic force and van der Waals become prominent for adhering on the substrate due to their possession of a large specific surface. To avoid the agglomeration between nano-particles, a dispersant is often added. However, for larger particles (> 1 µm) the specific surface area is reduced, which makes them non-interacting and non-agglomerating spherical solid particles in the liquid matrix (non-Brownian regime)^58^. This will reduce the interacting surface area between the substrate and the particles while creating a density mismatching dipping mixture. The insoluble spherical shaped hard inorganic particles (IP) have an average diameter of ~ 5 µm and particle to liquid density ratio > 7. Fast sedimentation of IPs will occur, which accumulates the IP at the bottom of the dipping vessel and creating a phase separation. To avoid sedimentation, the mixture is stirred before dipping for providing bulk motion aiding the dispersion and off-bottom suspension of IPs in LCS.

After dipping, we achieved an average of 129 µm coating thickness and we also observed a liquid bridge up to 1.5 mm after double-dipping which helped to fill a larger gap at nodes. For the epoxy specimen, a couple of gaps were large and the concentration of our epoxy glue was unable to fill them with the liquid bridge, as shown in Fig. 3. However, for the TLP specimen, only one node from the cell size 12 mm 2 × 2 × 2 structure was not filled by the liquid bridge. It should be noted here that, to provide better nodal contact for our TLP specimen, some struts are extended by ± 1.5 mm and the corresponding vertical struts become slanted, which can be seen in Table 1. The large gap TLP issue can be addressed by adding steel spheres (sphere 0.5 ~ 1.5 mm dia) into the LCS (composite interlayer) to match the wide gap in the green wire-shaped structure. The larger steel sphere will act as a space-filler to close the gap and create a bond-line similar to the medial axis in that the wide gap region compares to the traditional straight lines. Additionally, a large gap TLP joined (> 2 mm) has been reported by increasing the bonding temperature at about 130 °C^59^.

We also measured the weight of TLP specimens before and after dipping in dry conditions. A total of ~ 45% weight difference is observed, which is the weight of the glue and the powder combined as the solvent evaporated. As planned, more powder is seen to be entrapped between two adjacent surfaces in the liquid bridge volume. The remaining open surfaces are also covered with the powder coat due to polymer adhesive. The coated structure is sent to the vacuum furnace for solid-state TLP joining. The eutectic interlayer alloy on the faying surface melt at high temperatures (~ 1150 °C, which is below the substrate melting point), and the liquid phase fills the voids between the mating surfaces. As a result, high compressive load and strict surface finish are not necessary to cause diffusion in TLP bonding^60^. With proper process parameters (i.e., heating profile, time, gap) and interlayer materials (i.e., amount, composition), it can produce a very strong and interface free joint with no remnant of the bonding agent and with no or little surface preparation^61,62^. However, extensive diffusion can alter the substrate's microstructure, which can occur in the presence of a large amount of interlayer alloy^63^. We observed a 13% increase in wire diameter, which is the melt coat of the powder. This may cause weaker strut and low modulus of our lattice structure in our experiment. This issue can be addressed by optimizing the delivery volume of the IPs by designing the LCS. The delivery volume on the lattice surface can also be spatially controlled by surface modification techniques (i.e., etching) to control the interactions between the glue and the substrate.

**Figure 10:**
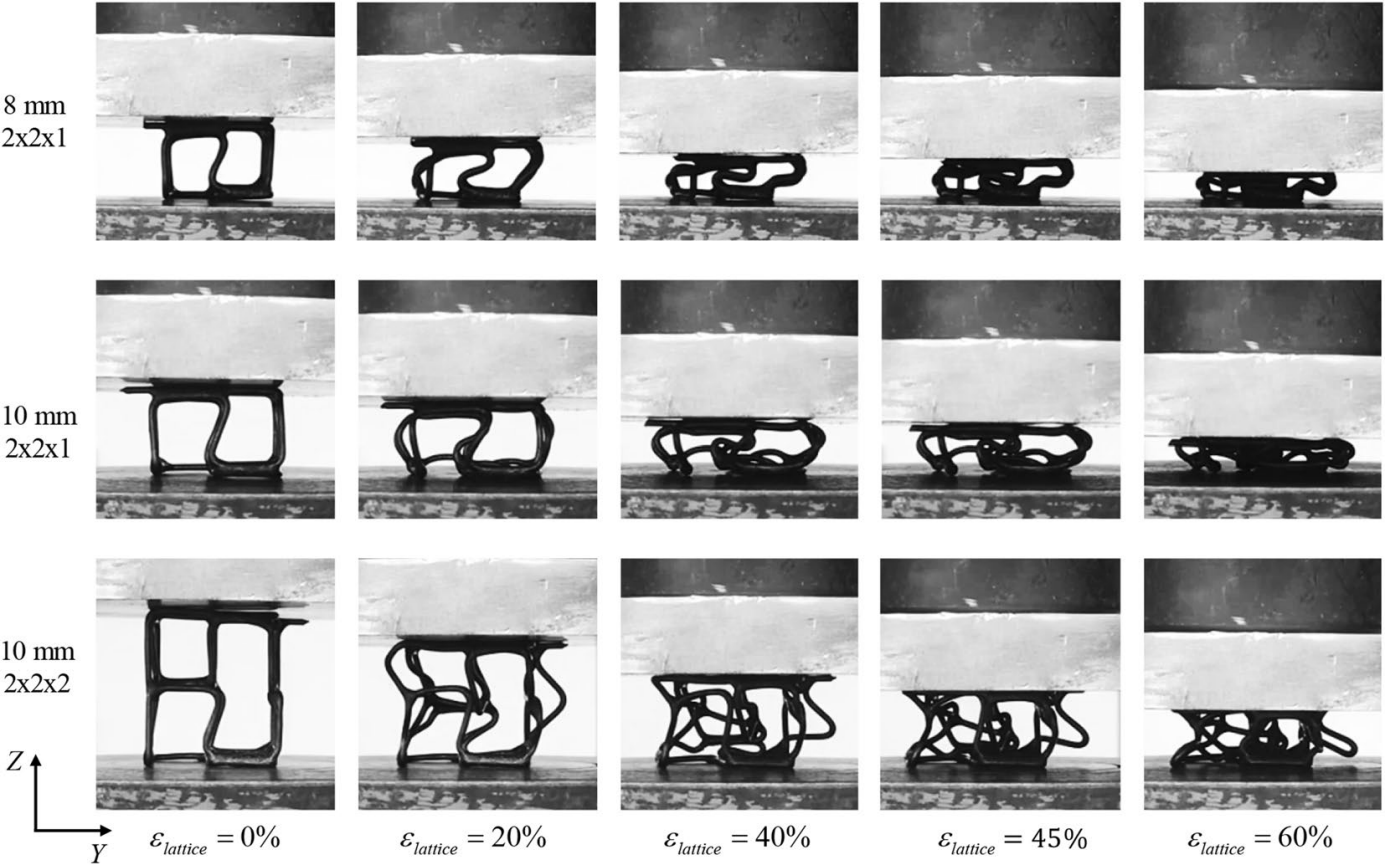
Compressing behavior of single stacked (2 × 2 × 1) 8 mm and 10 mm and double stacked (2 × 2 × 2) 10 mm cell size and lattice structures under Z-axis loading.

Pictures in Fig. 10 demonstrate the failure process of the 2 × 2 × 1 and 2 × 2 × 2 lattice structures subject to compression loading in Z-direction, at the overall lattice strains (${\varepsilon }_{lattice}$) of 0%, 20%, 40%, 45%, and 60%. For both lattice configurations at ${\varepsilon }_{lattice}=20\mathrm{\%}$, it can be observed that the deformation of structures predominantly occurred through inward and outward buckling of the vertical struts. As the load increases during the compression process (${\varepsilon }_{lattice}=40\mathrm{\%}$), the vertical struts buckled more and bent, and the horizontal struts also started to bend which is the expected phenomenon in bending dominated structures. Densification in single stacked 2 × 2 × 1 cell lattice starts approximately at 45%, 55%, and 63% strain levels for 8, 10, and 12 mm, respectively. For double-stacked 2 × 2 × 2 lattice, the plateau stress region still sustained up to 60% strain and densification is observed after 70% strain. This early densification for 2 × 2 × 1 c ell can be attributed to the cell design modification of slanted vertical struts explained earlier. Due to this additional angel, the vertical struts take a ‘S’ shape deformation, which introduces self-intersecting deformed members during compression. For smaller-sized single-stacking structures, this self-intersecting phenomenon causes densified walls, which are demonstrated earlier than other tested structures and caused early ‘pseudo’ densification behavior. However, this densification value is not far from a similar experiment reported in the literature. For example, stainless steel lattice is fabricated with the hollow truss by laying up collinear arrays with relative density from 3 to 23%^64^. They reported the compressive response exhibiting three regions: (a) elastic response (b) plastic yielding with plateau region and (c) densification. We also saw similar regions in our compressive plot of the structure (see Figs. 7,8, 9). Additionally, the densification strain has been reported at 50–60% which is comparable to our findings. This finding is also supported by the localized deformation mode with a higher inclination angle^65^.

We observed that in all samples, the strength and elastic modulus of the fabricated lattice decreases with the increase in cell size or reduced lattice relative density. This is in rapport with the traditional consensus of keeping the ratio of strut diameter and cell size close to 10. In the epoxy bonding, the failure occurs at the node as the cured material experience brittle failure. Whereas, in metal–metal TLP bonding, the strut of the structure deforms. The deformation of the strut is related to the size of the cell, i.e., small cell size with relatively softer material performs better in both bonding types than the larger cell type. For example, epoxy joined copper lattice (cell size 5 mm) has measured stress of 0.98 MPa, comparable to the metal–metal TLP joined AISI1006 steel (cell size 12 mm) lattice structure. We also observed anisotropy in our TLP joined structures, where we found lower mechanical properties in the X and Y direction load (Fig. 9). This can be attributed due to the lower number of vertical elements and with additional struts, the mechanical rigidity can be improved along those directions.

To further understand the characteristics of our TLP structures, we analyzed their failure mode and comparable performance. The young’s modulus for bulk AISI1006 is approximately 200 GPa which decreases with the topology along with their relative density. For cellular structures, the mechanical performance drops rapidly with decreasing relative density (increasing porosity)^66^. This loss of performance is often expressed with quadratic or higher-order scaling relationship between modulus and relative density. One such semi-empirical theory suggested by Gibson and Ashby^67^ is most commonly used to characterize the cellular materials. The power law and the strain of densification states below:

$$\frac{{E}_{latt.}}{{E}_{sol.}}=C{(\frac{{\rho }_{latt.}}{{\rho }_{sol.}})}^{n}$$

here, ${E}_{latt.} and {\rho }_{latt.}$ are, respectively, Young’s modulus and mass density of the lattice structure, whereas ${E}_{sol.}and {\rho }_{sol.}$ denotes the respective value of the constituent solid’s material property. Additionally, the decrease in performance has been attributed to constituent material properties, cell type (i.e., FCC, BCC, Cuboid, etc.)^24^ and arrangement of struts (i.e., number of struts joined in each node)^68^. The arrangement of the strut is often expressed via bending dominated vs stretch dominated structure by using Maxwell’s number^2^. However, in a recent publication by Chen *et. al*.^66^, a near-linear scaling of stiffness and relative density is achieved by the bending-dominated unit cells. We argue that the performance is also governed by the manufacturing modalities (i.e., investment casting vs. 3D printing vs. machining, etc.) which has rarely been differentiated. The degradation of performance from these various sources is often accounted for in the empirical constant such as $C \mathrm{and} n. \mathrm{T}$ he expected value of $n=2\sim 3$ and $C\approx 1$ for bending dominated (fixed joint) cell.

**Figure 11:**
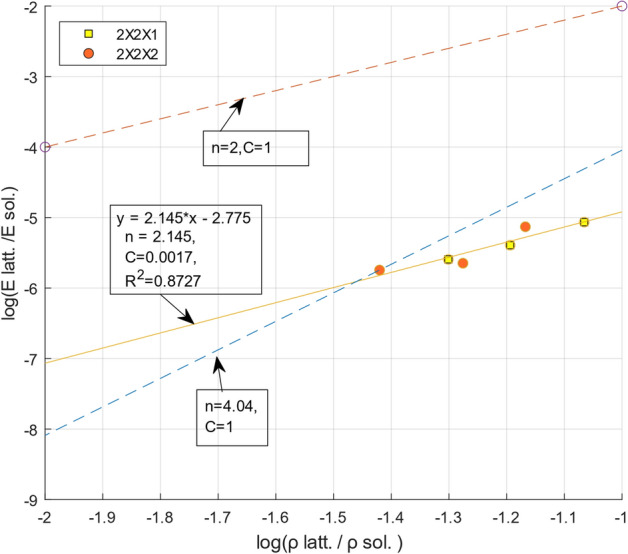
Scaling behavior of stiffness as a function of relative density.

By plotting the data of Tables 3 and 4, we determined their value with a fitted line as $n=2.145\mathrm{ and }C\approx 0.0017$ with the ${R}^{2}=0.87$. Two additional lines with n = 2 and C = 1, and n = 4.04 and C = 1 are drawn in Fig. 11 for comparison. The value of $C$ from our experiment is orders of magnitude lower than the suggested value, which is due to the low Young’s modulus. Rather than indicating that our experimental results are erroneous, this result simply highlights the limitations of capturing the loss of performance with such a simplified relationship and the empirical constants. Both cell geometry, materials, and manufacturing processes can be attributed to the lower value of $C \mathrm{and} n.$ For example, the relative density for our six samples ranges from 5% to 8.6%, which does not cover a good range of relative density. Thus the trend line of the data may not represent the characteristics of the lattices as the data points are very close to each other, shown in Fig. 11. Furthermore, our bending-dominated cuboid cell type has a larger cell size to strut diameter ratio (~ 8, 10, and 12) compare to commonly reported cell size ratio in the literature. For example, 3 mm bcc cell^69^ and 4 mm Gyroid cell^59^ were reported with 22% and 15% relative density, which are significantly different than our proposed structures. Strut-based topologies^70^ have been expressed by Maxwell's number which analytically determines the minimum strut number to equilibrate the external force and moment at the nodes. In axial compression load, the moments induced at the nodes are not equilibrated in the cuboid structure causing a bending dominated behavior. This phenomenon makes them more compliant and result consistent deformation than stretch dominated structures (i.e., BCC, FCC, Octet etc.)^71^ which may also cause the variation in results.

Additionally, our structures are built with softer annealed AISI 1006 materials compare to 3D printed 316 L and Al–Si10–Mg. Some struts in our structure are extended by ± 1.5 mm which makes them slanted and increases the nodal contact. Such design modification helps provide better nodal contact but will facilitate the localized deformation with this added inclination angle to the column lowering the compressive strength^65^. The presence of excess interlayer ink has been reported to deteriorate the strength of the wires^48^ and post-treatment for strength restoration is often necessary. However, no post-treatments are performed on our structures, which may provide room for improvement in our results. We also observed that a couple of the nodes are not connected due to bigger gaps. With such imperfection even with a small number of disjoined nodes, the performance can drop rapidly^24^, which may also contribute to the low compressive strength. As per the strength-density Ashby plot^72^, the compressive strengths of the fabricated wire lattice structures fall in the range of metal foams, with significant room for improvement. This identifies that our proposed wire lattice structures as promising candidates for the lightweight metallic cellular structure manufacturing process.

This paper demonstrates the rapid insertion of known materials in the form of functional multi-layer metallic lattice structure not yet possible with conventional tools. Hence, it accelerates the discovery of a novel functional and resource-efficient (lowering material cost, post-processing cost, and time) manufacturing modal. The use of readily available 1D wire materials compared to lower dimension feed-stock materials (powder or melt-pool) is a further innovation of this research. Metal wires are readily available, 90% cheaper than powder form, can survive the corrosive environment, contain lower contaminant, and shows homogeneous properties. Metal wires have been successfully used to fabricate periodic metal lattice structures using weaving techniques. The spin-insertion of helical preformed wires from multiple directions can construct the 3D structure with a reported relative density of about 3.5% after joining^48^, which is comparable to our proposed technique. However, the wire woven process is limited in geometries due to the required symmetry for spin-insertion. During the spin insertion, the preformed wire may experience higher order frictional resistance, which can cause the failure of wires (break, tear, etc.). As a result, the process is often restricted to the cylindrical wire form. Specialized equipment and tooling are necessary to automate the weaving of desired cell geometry due to multiple arrangements of preformed wire groups and complex motions. For example, the preformed elastic wires need to be fixed with external frames to prevent spring-back. Furthermore, the process may require both inplane and out-of-plane assembling for 3D lattices, which introduces an additional manufacturing complexity level.

The preformed shape of the wires introduces curviness in the struts of the 3D structure made from wire woven, which facilitates the bending dominated failure and deteriorates the strengths and moduli of the wire-woven metals. The performance between 3D wire weaving and the proposed technique can be compared using the experimental result reported in the literature^73^. The Wire-woven Bulk Cross (WBC) structure in the literature is compared with the cuboid structure shown in Table 5. However, the AISI 304 is used to fabricate the WBC structure compare to our AISI 1006. The reported relative density and slenderness ratio is also different than our tested result. Given both structures are bending dominated and slanted, the results shown in the table are comparable.

**Table 5:** Performance comparison between wire-weaving and proposed wire-bending technique.

Structure type materials	T-type WBC			Cuboid (AISI 1006)					
				2 × 2 × 1			2 × 2 × 2		
	AISI 304			AISI 1006			AISI 1006		
Relative density	11	8	5	8.6	6.4	5.0	6.8	5.3	3.8
Slenderness ratio	0.146	0.121	0.096	0.125	0.100	0.083	0.125	0.100	0.083
Compressive stress (MPa)	6.2	5.0	3.0	3.15	1.58	1.07	2.14	0.93	0.81

Additionally, lattice structures with controllable spatial properties (i.e., stiffness, density, filtration, etc.) are highly desirable for a precision-based application environment, but may only come with heterogeneous or aperiodic cellular structures^7^. Aperiodicity in lattice structure can be achieved with one or more of the three configurations (i) lattice pattern (e.g., BCC, Cubic, hexagon, etc.); (ii) shape conformity (e.g. conforming the object shape) and (iii) progressivity (e.g. gradient through size, shape or pattern of lattice) along *R*^3^ domain. It is the general consensus that optimal multi-functionality requires aperiodic lattice architecture but their realization itself is a challenge with current lattice manufacturing techniques including the wire-woven process. The proposed technique can construct an aperiodic lattice structure by bending the wire with the desired pattern and joining the nodes, opening up a new possibility.

The research proposed here is a novel multi-scale lattice structure manufacturing platform that will utilize the dip-coating science for delivering the interlayer particles in hard to reach places and liquid-bridge the gap for TLP bonding. Since its invention in the early 1970s, much work has been performed on optimizing TLP bonding parameters, a list that includes temperature, holding time^74^, pressure, base materials^75^, interlayer composition^76,77^, MPD types, diffusion rate^78^, and microstructure development^79,80^. However, faying surfaces are commonly assumed to be semi-infinite flat substrates and the interlayer material is generally applied in the form of thin foil^81–83^, fine powder (with or without binder)^84,85^, paste^86^, electroplate^87,88^ and, sputter^89,90^. Due to the intricate architecture of the lattice structure made with continuous wire, the faying surface will be inaccessible to traditional interlayer coating techniques. Besides, the structure will have wider gaps compared to the *status quo* TLP gaps. Dipping the intricate lattice structure in the interlayer particulate suspension as a delivery technique is the novelty of this work.

The knowledge presented in this paper may open-up a new avenue of advanced manufacturing systems that will actively integrate design (virtual), data (intelligence), material, and manufacturing (realization) processes to bring open-cell lattice structures into regular use. The process has the potentials to fill the ‘opportunity void’ in the ‘Ashby chart’. The proposed multi-scale lattice structure manufacturing platform will open up a new avenue for smart structures research. The proposed method will virtually generate zero waste and can bring environmentally conscious lattice part building techniques and thus may help to build ‘greener’ products and processes.

The generated structure can be suitable for impact-absorbing structures^91^ for microelectronics, medical devices; tissue scaffolds; shelters; porous orthopedic implants; reinforcement architecture, shape morphing^92,93^, cores with outer skin-wall^53^ or net/twin skin^94,95^ objects. Other uses include aircraft components such as fuselage and wings, automobile components such as doors, dashboards micro drones (artificial insects), dwelling/storage units, boat hulls and components, and many other structures. The advantage of such large structures, such as dwellings/storage units is that the wire components and the covering materials can be delivered separately and assembled on-site, such as at the site of a natural disaster or military outpost. This would make the delivery easier and more efficient than the traditional finished structures.

## Method

### Joining the loose nodes with polymer epoxy

**Figure 12:**
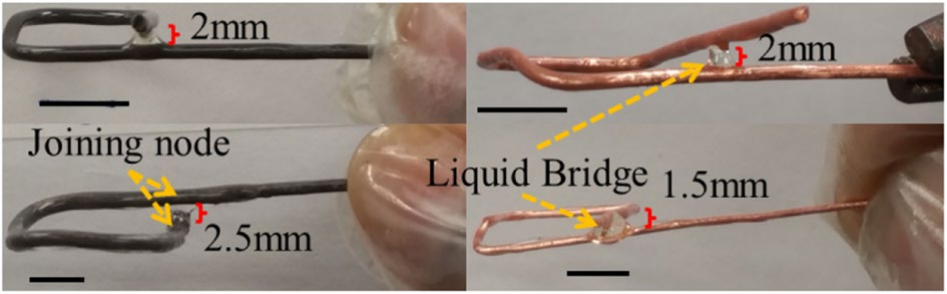
Liquid bridge formation in the bended wire node with carbon steel (dia:1.63 mm) and copper (dia: 1.02 and 0.51 mm). Scale bar 1 cm.

For adhesive, commercially available 2000 Epoxy Resin is used with 2120 Epoxy Hardener (mix ratio by volume: 3:1 with a viscosity of 950–975 cps at 77 °C and density 0.041 lb/in^3^). Before dipping the loose lattice structure, they are degreased with isopropyl alcohol and trichloroethylene bath followed by surface etching for improved adhesive bonding. The bent lattice structures are dipped in the epoxy resin orthogonally for 30 to 60 s and then placed in an oven for 30 min at 100 °C. The prepared epoxy solution can bridge a range of gaps that are common in the loose lattice structure as shown in Fig. 12.

### Node joining with transient liquid phase (TLP)

#### Preparation of liquid carrier delivery system

**Figure 13:**
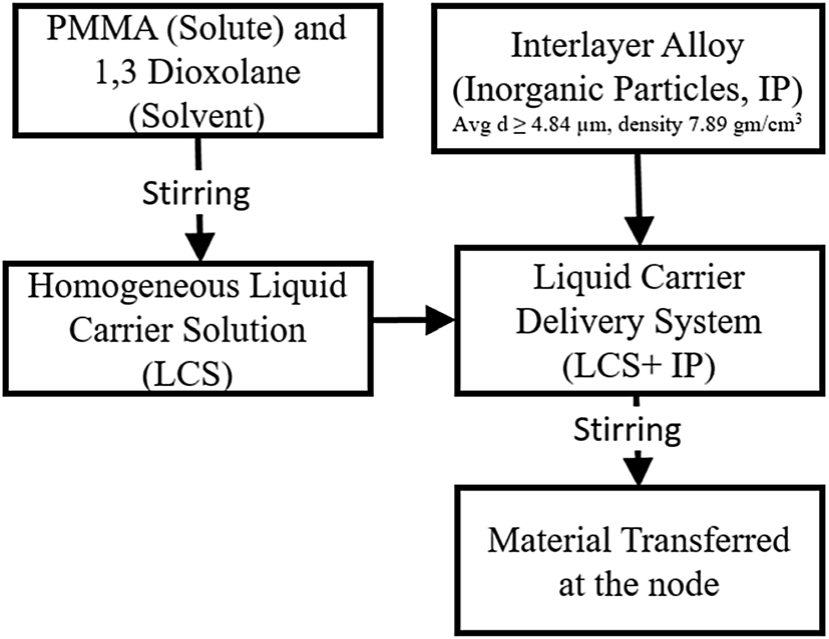
Flowchart of interlayer material suspension preparation.

Transient liquid phase (TLP) bonding is a metal–metal joining process that combines the characteristics of both diffusion bonding and brazing. TLP requires putting an interlayer (filler) metal/alloy containing a melting point depressant (MPD) of the substrate metals between the faying surfaces. Nicrobraz powder (Wall Colmonoy company, Ohio) is considered as the inorganic particles (IP), which need to be transferred to the node of the loose lattice. These powders are usually large-sized (Avg d ≥ 4.84 µm, density 7.89 gm/cm^3^, liquidus temperature 950 °C) spherical hard particles and commonly used for joining similar or dissimilar material using various solid-state joining process (e.g., diffusion bonding, transient liquid phase bonding, and brazing). Due to the size of the unit cell and complex structure, the nodes are not easily accessible within the loose lattice. A liquid carrier system is designed to deliver the large, hard inorganic particles at the node, as shown in Fig. 13.

#### Solute/binder

Polymethyl methacrylate (PMMA) is considered as the binder polymer. It is a popular transparent acrylic resin commonly known as plexiglass. This amorphous thermoplastic demonstrates satisfactory gap-filling properties, excellent thermal stability (flash point > 250 °C) and tensile strength (72 MPa), low density (~ 1.17 g/cm^3^), relatively harmless non-explosive and non-flammable material. The popular aphorism of “like-dissolve-like” governs its solubility^96^.

#### Solvent

Toluene, chloroform, butanone (MEK), and Dioxolane are some common solvent for PMMA. Among them, 1,3 Dioxolane has a favorable toxicity profile, low density (~ 1.06 g/cm^3^), and an excellent evaporation rate. This cyclic acetal facilitates polymerization of PMMA, has an intermediate polarity (dielectric constant = 7.13), and vapor pressure 70 mmHg (20 °C). Both solute and solvent are purchased from Sigma Aldrich and stirred for 8 h to make the *Liquid Carrier Solution (LCS)*. About 40% (v/v) inorganic particles are added to the solution before dipping. Due to the density mismatching between IP’s (7.89 gm/cm^3^) and LCS solution will facilitate fast sedimentation of IP’s and dragging them to the bottom. A magnetic stirrer is used to disperse the particles spatially. The loose lattice is dipped in the ‘pseudo’ suspension, and the particles are transferred at the node.

#### Optimizing the composition of liquid carrier delivery system

The volume concentrations of binder (PMMA), interlayer alloy (Nicrobraz 51 powder), and solvent (1,3-Dioxolane) is considered as the three factors for the experiment. From a preliminary experiment it was found that the usable volume concentration domains of these three constituents for the dip coating process are 2.13–7.5%, 29.75–52.5%, and 40–66%, respectively. At first, three concentration (%v) levels (40%, 53%, and 66%) of the solvent (1,3-Dioxolane) in the suspension were chosen. The remaining of the mixture was constituted with PMMA and Nicrobraz 51 powder at 1:7, 1:11, and 1:15 volume ratios. The naming convention of the mixture is as follows: “D40-P1-N7” indicates that the volume fraction of Dioxolane (D) is 40% and the volume ratio of PMMA (P) and Nicrobraz 51 (N) is 1:7.

**Figure 14:**
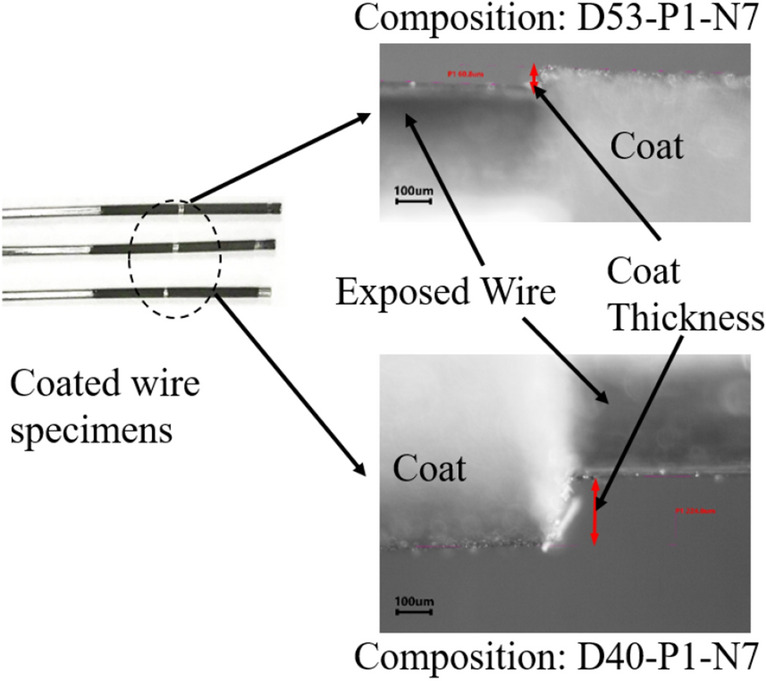
Micrographs of coated wire and coating thickness measurement for composition D53-P1-N7 (top) and D40-P1-N7 (bottom).

**Figure 15:**
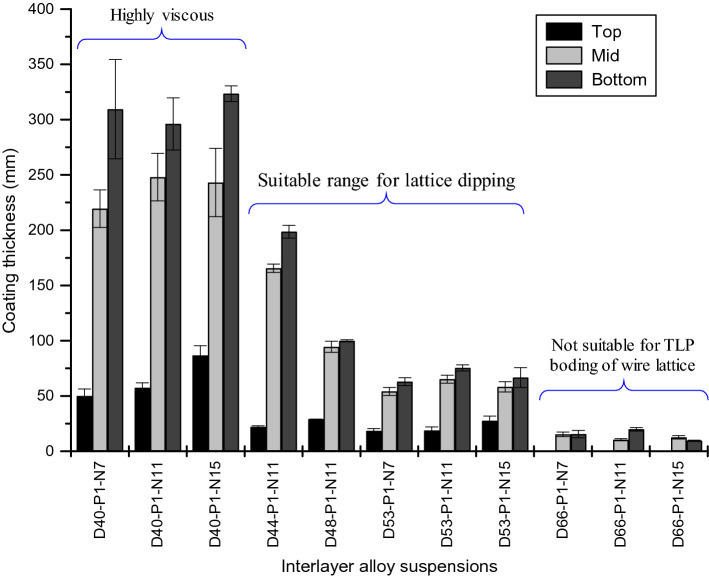
Coating thickness at three different locations (top, mid, and bottom) of the wire specimens dipped into the prepared interlayer alloy suspensions.

To determine the workable composition, three wire specimens (three replicates) are cleaned and dipped in each composition, and the thickness is measured using a microscope, as shown in Fig. 14. AISI1006 wires with a diameter of 1.02 mm ± 0.02 mm are cleaned successively with 400, 600, and 1200 grit emery papers. Then the wires are cut into 6 cm ± 2 mm straight specimens with a high-speed precision cutter (Allied High Tech Products, Inc). The wire specimens were ultrasonically washed in acetone and air dried for the dipping process. The coated specimens were kept in the open air at room temperature overnight to let the coating completely dry. Some wire specimens coated with D40-P1-N7 and D53-P1-N7 composition are shown in Fig. 14. The coating thicknesses are measured from the optical micrographs at three locations (top, mid, and bottom) which are represented with a column chart in Fig. 15.

Composition D40-P1-N7, D40-P1-N11, and D40-P1-N15 are highly viscous due to the lower concentration (40%) of solvent and create a significantly thicker coating. The resulting yield stress may deform the green (loose) lattice structure during the dipping process. On the other hand, composition D66-P1-N7, D66-P1-N11, and D66-P1-N15 have lower viscosity and the coating thickness is low. As a result, the volume of interlayer material transferred is insufficient for the TLP joining. Based on these results, two additional compositions D44-P1-N11 and D48-P1-N11 are also tested and their results are added in Fig. 15. We used composition D44-P1-N11 to join the lattice shown in Table 1.

#### Node joining in the vaccum furnace

At high temperature (below the substrate melting point), the interlayer alloy liquefies and fills the joint area by capillary action. This liquid phase forms a capillary bridge in the interlayer region. Bonding then occurs as inter-diffusion changes the local composition, causing it to solidify isothermally. The temperature is raised to 580 °C at a rate of 20 °C/min and held there at 580 °C for 20 min. The polymer binder (PMMA) volatilizes at this temperature and leaves no residue in the vacuum furnace. The temperature is then raised to 1150 °C at a rate of 20 °C/min and held it there at 1150 °C for 1 h and 45 min. After that, the specimens are furnace cooled. After furnace cooling, the maximum gap sizes filled by melted and diffused interlayer alloy.

#### Mechanical testing

The Fabricated and joined AISI1006 wire lattice structures are tested under compression loading to establish their stress–strain behavior. The compression tests are performed with an Instron 5567 universal testing machine utilizing a 30 kN load cell. The crosshead speed applied during the tests is 3 mm/min. The tests are controlled and the data are recorded using Bluehill software for Instron. All the tests are recorded with a high-speed camera and pictures at different strain levels are snapped from the video to analyze the deformation and progressive failure mechanism of the lattice structures. The tests are performed for compression loading on three principal directions (*X*, *Y*, and *Z*) to capture the mechanical anisotropy of the specimen structures shown in the “Result” section. The testing specimens are fabricated with AISI1006 low carbon steel wire of diameter 1.02 mm.

## Conclusion

There is a disconnect between design, geometry, process, and data for the fabrication of the functional 3D lattice structure. In this paper, a novel metal lattice manufacturing process has been demonstrated with wire material. The proposed method is able to construct a range of unit lattice, including cuboid, pyramidal, hexagon, truss-like, prismatic, BCC, and FCC. The mechanical properties of the structures can be improved with better design, bending accuracy, optimum dipping mixture, particle size, which can be the future direction of this research.

The proposed methodology demonstrates the feasibility of making a metal wire structure from a continuous rod. For implementation, we activate a semi-automated process-plan for bending the wires where 2.5 D layers are bent with our lab-developed programmable machine. These layers can be stacked to realize the 3D shapes. The degree of automation can be increased and improved by deploying collaborative multi-robotic assembly and the error can be minimized by incorporating artificial intelligence in the future.
